# A Spatiotemporally Coupled Carbon Flux Monitoring System for Salt Marsh Wetlands Based on Integrated Land–Air Collaborative Intelligence

**DOI:** 10.3390/s26102966

**Published:** 2026-05-08

**Authors:** Yichen Zha, Zeyan Wang, Jianping Shi

**Affiliations:** School of Electrical and Automation Engineering, Nanjing Normal University, Nanjing 210023, China; 21230625@njnu.edu.cn (Y.Z.); 21240808@njnu.edu.cn (Z.W.)

**Keywords:** salt marsh wetlands, carbon flux monitoring, multi-algorithm fusion technology, DNDC model

## Abstract

Against the backdrop of intensifying global climate change, reducing carbon emissions has become a shared global objective. Blue carbon, as a significant carbon sink type, still lacks a mature assessment framework. Monitoring carbon fluxes in marine salt marsh wetlands is a core technology for accurately evaluating blue carbon potential. In response, this study independently developed a spatiotemporally coupled carbon flux monitoring system for marine salt marsh wetlands. The system consists of real-time monitoring equipment, a cloud-based intelligent storage and visualization analysis platform, and a terminal assessment system. It enables the real-time monitoring of carbon fluxes across multiple spatial scales and integrates time-series patterns to assess carbon sequestration potential from multiple dimensions. To address the bottleneck of sensor accuracy, a multi-algorithm fusion technology was innovatively developed, significantly enhancing the accuracy of monitoring data. A modular integrated design was employed to construct a land–air integrated monitoring architecture, which is adaptable to the complex environments of salt marsh wetlands. This facilitates long-term automated monitoring while reducing the need for manual intervention. The terminal assessment system processes spatial-scale data using the DeNitrification-DeComposition model (DNDC 9.5) and integrates time-series carbon flux patterns, enabling precise quantification of marine carbon sink potential through spatiotemporal comprehensive analysis. The system first completed performance verification during the experimental phase, acquiring a total of 5760 sets of valid monitoring data, with a data qualification rate of 99.72%. The proposed multi-algorithm fusion method kept monitoring data fluctuations within 0.5%, and the relative error of the spatiotemporal integrated prediction was as low as 0.31%, thereby ensuring the stability and accuracy of long-term in situ monitoring. Based on this, a one-year field validation was conducted in a 100-hectare coastal salt marsh wetland in Dafeng, Yancheng. Using a spatiotemporal coupling assessment, the annual total carbon sequestration of this area was estimated at 1498.4 tons of carbon, with an assessment error of only 5.1%, achieving precise quantification of the blue carbon sink in the salt marsh wetland. This study provides reliable technical support for evaluating the carbon sequestration capacity of coastal salt marsh wetlands, contributing to the implementation of carbon emission reduction strategies. It also offers a scientific basis for global carbon cycle research and carbon sink management decision-making.

## 1. Introduction

### 1.1. Research Background

Global warming is intensifying, making “carbon sinks” a pivotal element in climate governance. The ocean, as the largest active carbon reservoir, absorbs approximately 26% of anthropogenic CO_2_ emissions. Among marine systems, salt marsh wetlands are a core component of blue carbon. Their annual carbon sequestration per unit area reaches 1500 g/m^2^, which is 5 to 10 times that of tropical rainforests, granting them a significant role in the global carbon cycle [1]. Lu et al. identified salt marshes as one of the most significant natural carbon dioxide sinks, serving as a vital component of biological carbon storage on Earth due to their exceptionally high primary productivity [2]. Ge et al. further proposed that coastal wetlands play a critical role in regulating atmospheric CO_2_ concentrations, thereby contributing substantially to climate change mitigation [3]. Therefore, accurate monitoring and scientific prediction of carbon fluxes in salt marsh wetlands are of great importance for climate regulation and the achievement of carbon neutrality goals.

However, current monitoring equipment is prohibitively expensive; for instance, LI-COR series instruments can cost over one million yuan per unit. These systems also involve complex maintenance and exhibit poor adaptability. The harsh environmental conditions of salt marshes, characterized by high salinity, high humidity, and tidal fluctuations, cause existing equipment to suffer from data inaccuracies, high failure rates, and a need for frequent manual intervention. Consequently, they are ill-suited for meeting the demands of routine, long-term monitoring.

### 1.2. Related Work and Problem Analysis

Monitoring of carbon fluxes in salt marsh wetlands has established mainstream approaches, including eddy covariance and static chamber–gas chromatography methods. For instance, Mayen et al. employed eddy covariance technology to investigate atmospheric CO_2_ exchange in temperate salt marshes [4], while Stolpmann et al. quantified carbon fluxes in estuarine salt marshes using static chamber–gas chromatography [5]. The Soares team integrated Landsat satellite imagery and high-resolution remote sensing data with cloud computing to analyze global salt marsh changes and estimate carbon emissions [6]. Additionally, Barkley et al. accurately retrieved atmospheric carbon dioxide concentrations from satellite spectral data by applying differential optical absorption spectroscopy [7].

The assessment model framework for carbon sequestration in salt marsh wetlands is becoming increasingly mature. The Waquoit Bay monitoring station in the United States developed flux characteristic indicators for salt marshes through dimensional analysis [8]. Meanwhile, a greenhouse gas flux prediction model created by a monitoring station in Zhejiang Province achieved Nash–Sutcliffe efficiency values ranging from 0.80 to 0.91, demonstrating a favorable predictive performance [9]. Biogeochemical models such as DNDC (DeNitrification-DeComposition) are widely employed to simulate carbon and nitrogen cycles in salt marsh wetlands. Process-based models have been coupled with machine learning algorithms, and Natel et al. further developed a high-spatiotemporal-resolution carbon flux estimation model, which reduces estimation uncertainty [10].

However, the characteristic high-salinity and high-humidity conditions of salt marsh wetlands significantly compromise the measurement stability and accuracy of gas sensors. Xu et al. demonstrated that environmental humidity and salt spray induce substantial interference in nondispersive infrared (NDIR) CO_2_ sensors, resulting in measurement drift and reduced precision [11]. Demik et al. further confirmed that fluctuations in humidity cause baseline drift and response attenuation in sensors, directly undermining the reliability of carbon flux monitoring data [12]. Consequently, existing studies on carbon flux in salt marsh wetlands have struggled to mitigate the profound impacts of these environmental factors on sensor performance.

Moreover, traditional approaches to carbon flux monitoring, which typically rely on short-term field sampling followed by offline laboratory analysis, are inadequate for capturing the true dynamic characteristics of wetland carbon fluxes. Wang et al. noted that the static chamber–gas chromatography method, dependent on manual sampling and subsequent laboratory analysis, suffers from significant time lags and spatial gaps [13]. In salt marsh wetlands, this approach may fail to capture the driving effects of transient environmental changes, such as tides and temperature, on carbon fluxes. Chen et al. further demonstrated that offline analysis introduces sampling errors and time delays, potentially leading to deviations of up to 40% in carbon flux estimates, thereby severely compromising data reliability and spatiotemporal continuity [14]. Consequently, current mainstream monitoring approaches generally face limitations in achieving in situ, real-time, and synchronous monitoring and data analysis, exhibiting clear deficiencies in both spatiotemporal continuity and data reliability.

Current monitoring and assessment of carbon fluxes in salt marsh wetlands still face common challenges. Mainstream monitoring equipment is costly and imposes stringent site requirements. General-purpose devices are difficult to adapt to the unique high-salinity, high-humidity, and tidal alternating environment of salt marshes, leading to issues such as corrosion and data distortion. Moreover, mainstream assessment models lack localized parameter optimization for salt marsh wetlands, resulting in insufficient regional adaptability.

Overall, three core and longstanding challenges persist in this field. First, the monitoring system is incomplete, lacking an integrated solution that combines large-scale coverage, high-precision data collection, and real-time transmission. This results in spatiotemporal coverage gaps and low efficiency in multi-source data fusion. Second, assessment methods remain singular, failing to achieve coupled analysis of carbon fluxes across spatiotemporal scales. Consequently, accounting accuracy often falls short of practical requirements. Third, there is inadequate equipment adaptability and technology application. The development of specialized monitoring devices lags, and the application of technologies such as multi-sensor data fusion is not yet mature, which severely constrains the scalable enhancement of blue carbon monitoring capabilities.

### 1.3. Research Contributions and Paper Structure

To address the aforementioned research gaps, this study independently developed a spatiotemporally coupled carbon flux monitoring system for coastal salt marsh wetlands. Its core contributions are reflected in innovations across the following three aspects.

The first aspect is technological innovation. A multi-sensor data fusion technique integrating Gaussian Process Regression (GPR) and Gated Recurrent Units (GRUs) is proposed, which reduces the fluctuation range of environmental monitoring data to 0.5% and significantly enhances data accuracy.

The second aspect is methodological innovation. A spatiotemporally coupled carbon sink assessment framework was constructed based on the DNDC model. This framework integrates spatially distributed monitoring data with temporal evolution patterns, addressing the limitations of single-scale assessments and improving the precision of carbon sink accounting. Third, the architecture is innovative: a land–air integrated collaborative monitoring framework specifically designed for salt marsh wetlands is developed. This framework incorporates intelligent lifting devices, a hybrid wind–solar power supply system, and unmanned aerial vehicle (UAV) swarm technology, enabling long-term automated monitoring in complex environments and significantly reducing the need for manual intervention.

The execution of this study effectively addresses the technological gap in quantifying key carbon flux processes. It provides reliable data support for decision-making related to carbon flux trading, ecological restoration, and climate governance. The developed monitoring system achieves multi-dimensional optimization in terms of precision, adaptability, and cost-effectiveness. It contributes to the advancement of global carbon cycle research and the implementation of climate governance practices, offering crucial technical assurance for the scientific development and sustainable management of blue carbon resources in salt marsh wetlands. The subsequent sections of this study are organized as follows. Section 2 details the research materials and methods, introducing the monitoring system design and core technologies such as multi-algorithm fusion and integrated land–air monitoring. Section 3 presents the experimental validation results, including sensor data, optimization of UAV sampling points, and carbon sink predictions from the DNDC model. Section 4 compares this study with traditional methods and similar equipment, analyzing its core innovations. Section 5 summarizes the key contributions, acknowledges limitations, and outlines directions for future work.

## 2. Materials and Methods

As previously discussed, coastal salt marshes serve as critical blue carbon reservoirs. However, current blue carbon monitoring technologies remain in their infancy, with non-standardized accounting methodologies presenting significant challenges. The unique high-salinity, high-humidity, and tidal-influenced environment of salt marshes readily leads to corrosion and data inaccuracies in general-purpose monitoring equipment, necessitating frequent manual intervention. High-precision equipment is costly and complex to maintain. Existing monitoring efforts predominantly rely on single-point stations, resulting in spatial and temporal coverage gaps. Carbon sink assessments lack spatiotemporal coupling analysis, leading to high result uncertainty and hindering the scaling up of blue carbon monitoring.

**Figure 1:**
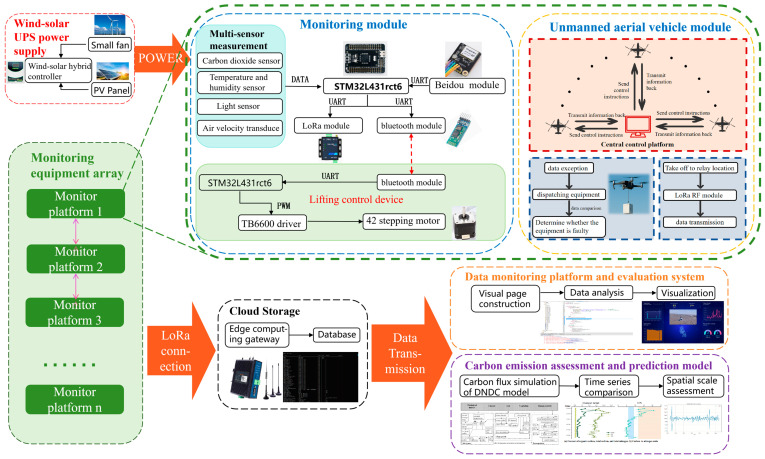
Schematic diagram of the integrated wetland environmental monitoring and carbon assessment system. The system comprises: the wind–solar power supply subsystem, distributed multi-sensor monitoring nodes, unmanned aerial vehicle-assisted equipment dispatching module, cloud data aggregation gateway, and the data visualization and DeNitrification-DeComposition model-based carbon emission evaluation platform.

To address these bottlenecks, this study developed a spatiotemporally coupled carbon flux monitoring system for coastal salt marshes, achieving breakthroughs in key technologies. A multi-sensor data fusion framework integrating Gaussian Process Regression (GPR) and Gated Recurrent Units (GRUs) was constructed, controlling data fluctuation within 0.5%. A land–air integrated collaborative monitoring architecture was designed, enabling intelligent monitoring through the coordination of unmanned aerial vehicles (UAVs) and ground-based intelligent lifting equipment. Finally, a spatiotemporally coupled carbon sink assessment system based on the DNDC model was established, integrating spatially distributed monitoring data with temporal evolution patterns. The system design process, shown in Figure 1, is divided into the following three parts, which will be detailed below: Section 2.1, Section 2.2 and Section 2.3.

### 2.1. Carbon Flux Monitoring Equipment

The carbon flux monitoring equipment employs a modular architecture designed for adaptability to the complex environment of coastal salt marshes. It achieves long-term, precise, and automated environmental data collection and transmission through the coordinated operation of multiple modules. The core system comprises three primary functional modules: an intelligent lifting monitoring device based on seamless positioning technology and multi-sensor measurement, an edge intelligent computing gateway module based on machine learning, and a wind–solar hybrid power supply system utilizing an Uninterruptible Power Supply (UPS).

#### 2.1.1. Intelligent Lifting Monitoring Device

**Figure 2:**
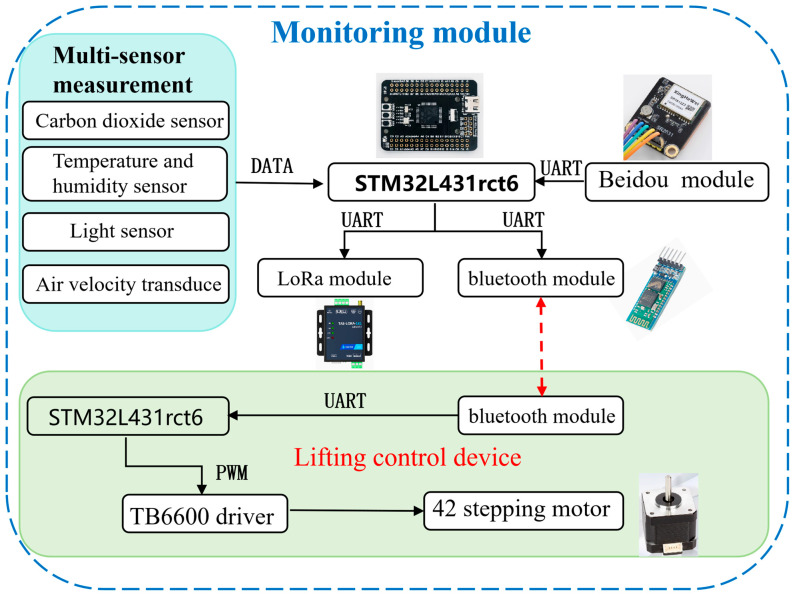
Schematic diagram of the intelligent lifting monitoring device. The device comprises two functional parts: the multi-sensor monitoring unit (with carbon dioxide, temperature-humidity, light, and air velocity sensors, Beidou positioning, LoRa and Bluetooth communication based on the STM32L431 microcontroller (STMicroelectronics, Geneva, Switzerland)) and the lifting control unit (controlling the 42-type stepping motor via the TB6600 driver (TASTEK, Shenzhen, China) under STM32L431 control).

Serving as the core for on-site data acquisition, this device consists of three coordinated sub-modules: a multi-sensor integrated data acquisition submodule, an intelligent lifting submodule, and a seamless positioning submodule. This design balances data accuracy, environmental adaptability, and monitoring continuity. The configuration is shown in Figure 2.

**Figure 3:**
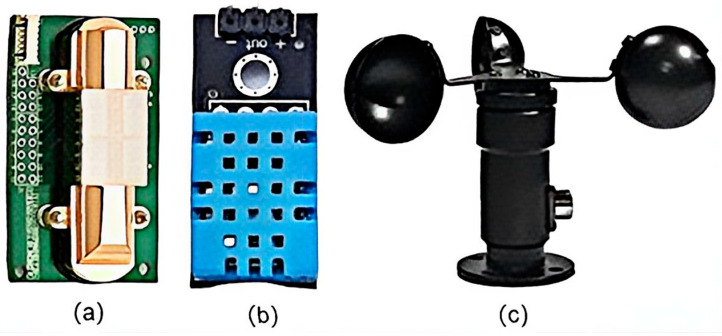
Some physical objects of sensors: (**a**) NDIR non-dispersive infrared CO_2_ sensor. (**b**) DHT11 temperature and humidity sensor. (**c**) Anemometer.

The first component is the multi-sensor integrated data acquisition submodule which employs a set of high-precision sensors treated for corrosion resistance. Its core component is an NDIR (Non-Dispersive Infrared) CO_2_ sensor (Winsen, Zhengzhou, China), which monitors key carbon flux data based on the exclusive absorption band at 4.26 micrometers. This sensor features a measurement range of 0–5000 ppm, a resolution of ≤1 ppm, and effectively resists interference from water vapor and salt spray [15]. Additionally, the submodule integrates supplementary sensors including a DHT11 temperature and humidity sensor, a BH1750 light intensity sensor, and an anemometer to comprehensively capture critical environmental factors influencing carbon flux, with some sensors shown in Figure 3. All sensors are under the unified timing control of a STM32 board, with a default sampling rate of once every 5 s. The raw data undergoes preliminary filtering before being cached locally [16].

Prior to field deployment, the CO_2_ sensors underwent systematic calibration in a controlled laboratory environment to ensure the accuracy of in situ monitoring data. Calibration conditions were maintained at a temperature of 20 ± 2 °C and a relative humidity of 50 ± 5% RH. The procedure commenced with zero-point calibration, during which 99.999% high-purity nitrogen was introduced to the sensor for 10 min; once readings stabilized, the output was reset to zero. This process was repeated three times, and the average value was recorded. Subsequently, multi-point span calibration was conducted using seven standard CO_2_ gas concentrations: 0 ppm, 500 ppm, 1000 ppm, 2000 ppm, 3000 ppm, 4000 ppm, and 5000 ppm. For each concentration level, gas flow was stabilized for 5 min before recording output data, with three replicate measurements performed per point [17]. A regression analysis comparing sensor readings against standard gas concentrations revealed that measurement deviations across all concentration points remained below ±15 ppm, thereby satisfying the precision requirements for carbon flux monitoring in salt marsh wetlands. Furthermore, to ensure long-term monitoring stability, a portable 2000 ppm standard gas cylinder was employed for rapid on-site verification before and after each field experiment. If the deviation exceeded ±30 ppm, immediate recalibration was conducted to guarantee the reliability of data quality throughout the entire process.

The second component is the intelligent lifting submodule, which incorporates an adaptive lifting mechanism. Driven by an STM32L431 microcontroller, this mechanism operates a TB6600 driver (TASTEK, Shenzhen, China) and a 42-step stepper motor (Leadshine, Shenzhen, China) to achieve a lifting range of 0–1.5 m at a speed of 5 mm/s [18]. The height can be automatically adjusted based on water level and meteorological data. The lifting structure employs 304 stainless-steel guide rails and sealed bearings, providing IP67 (Ingress Protection 67) protection against water and dust. It is designed to operate in ambient temperatures ranging from –20 °C to 60 °C [19].

**Figure 4:**
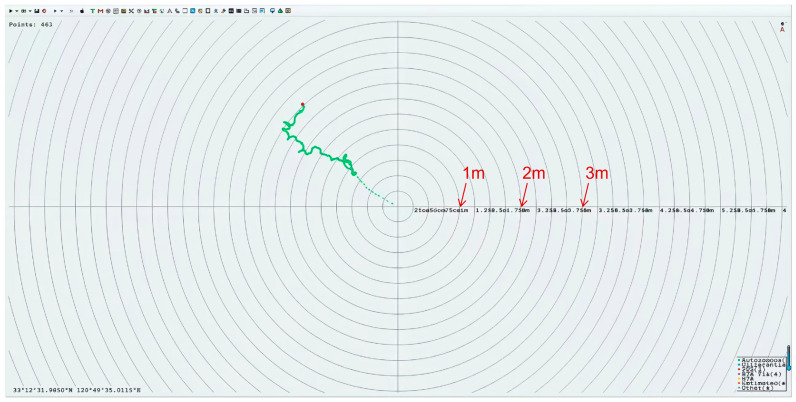
Positioning performance of the monitoring device in field test. The positioning test was conducted at the Dafeng Wetland in Yancheng, and the result shows that the device’s positioning error is less than 1 m, which meets the accuracy requirements for wetland environmental monitoring applications.

The third component is the seamless positioning submodule which incorporates the SR2631 BeiDou positioning module (BDStar, Beijing, China). Its performance is enhanced by an extended Kalman filter algorithm, achieving a positioning accuracy of ±1 m [20]. The module exhibits excellent signal reception sensitivity and a fast update rate, providing reliable spatial traceability for carbon flux data. This capability supports grid-based analysis in distributed networking. The positioning performance is shown in Figure 4.

#### 2.1.2. Edge Intelligent Computing Gateway Module Based on Machine Learning

**Figure 5:**
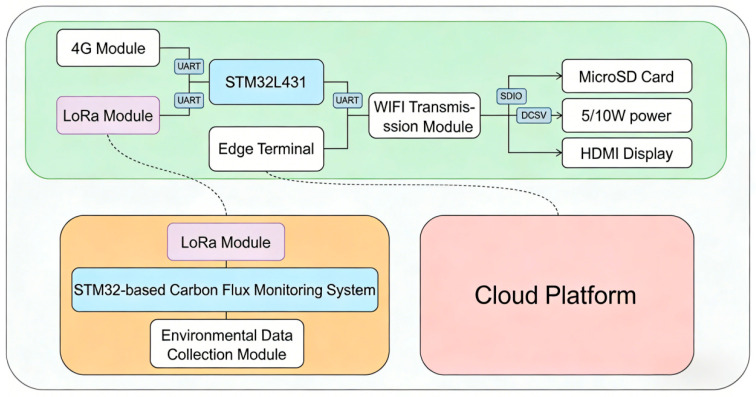
Schematic diagram of the edge intelligent computing gateway module. The gateway comprises an STM32L431-based control unit, which receives data from LoRa and 4G modules, processes it via the edge terminal, and transmits it to the wireless fidelity module. It supports local storage, display, and power management, enabling data interaction with the carbon flux monitoring system and the cloud platform.

The edge intelligent computing gateway serves as a hub for data preprocessing and transmission, performing local data cleaning, anomaly detection, and protocol conversion. Centered on the low-power STM32L431 main control chip, it incorporates a lightweight machine learning model to enable intelligent decision-making at the edge. The gateway collects raw sensor data via the Modbus-RTU protocol [21]. After removing invalid data through sliding window filtering and outlier detection, it employs a pre-trained GRU neural network model for imputation and prediction to enhance data continuity [22]. The processed data is then uploaded to the cloud via LoRa (Long-Range) or 4G (4th-Generation Mobile Communication Technology) modules, with local storage also supported. In the event of a network interruption, data is automatically cached and resumes transmission from the breakpoint upon recovery, ensuring no data loss. The specific workflow is shown in Figure 5.

#### 2.1.3. Wind–Solar Hybrid Power Supply System with UPS

**Figure 6:**
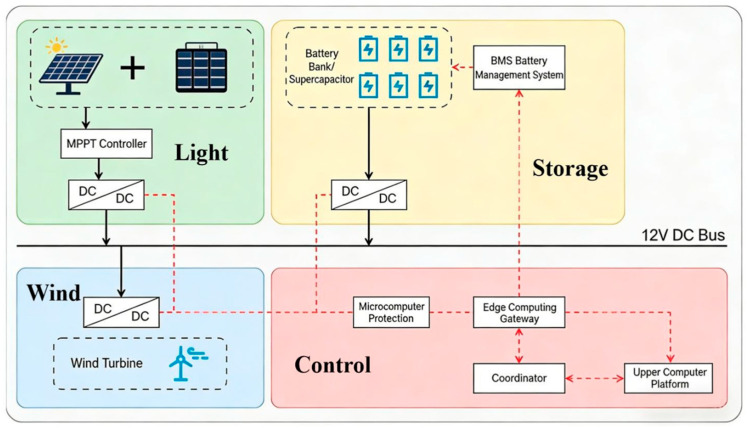
Schematic diagram of the wind–solar hybrid power supply system. The system comprises four functional units: the solar power generation unit, wind power generation unit, energy storage unit with battery management system, and control unit. It realizes off-grid power supply for the monitoring system through a 12 V direct current bus.

To meet the long-term unattended monitoring power demands in salt marsh wetlands, a wind–solar hybrid power supply system was designed and integrated with an UPS to achieve stable all-weather power provision [23]. The system comprises a 200 W monocrystalline silicon solar panel, a 100 W small vertical-axis wind turbine, a 12 V/100 Ah lithium iron phosphate battery bank, and an intelligent charge–discharge controller. It automatically switches power supply modes based on sunlight intensity and wind speed, prioritizing the use of renewable energy. During periods of continuous overcast weather or calm conditions, the UPS battery bank provides backup power, ensuring an operational endurance of no less than 72 h. The charge–discharge controller features multiple protection functions, enabling real-time monitoring of battery status and energy consumption. It transmits analytical reports via cloud push technology. With its integrated design, the power system offers easy installation and low maintenance costs, effectively addressing the power supply challenges for monitoring equipment in remote wetland areas. The configuration is shown in Figure 6.

### 2.2. Cloud Intelligent Storage and Visualization Analysis Platform

**Figure 7:**
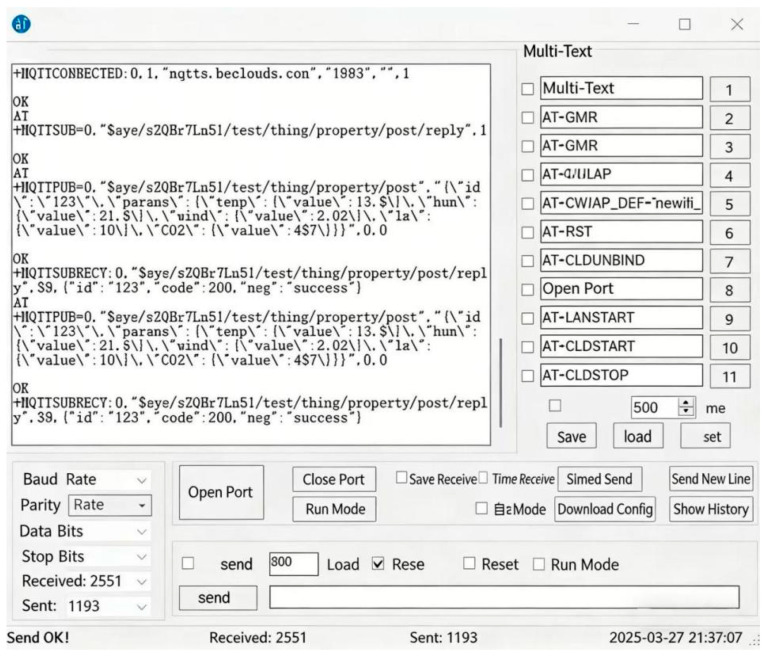
Serial port command interaction test of the monitoring device. The figure shows the serial port debugging interface, where AT commands are used to send control instructions to the monitoring device and receive environmental monitoring data.

**Figure 8:**
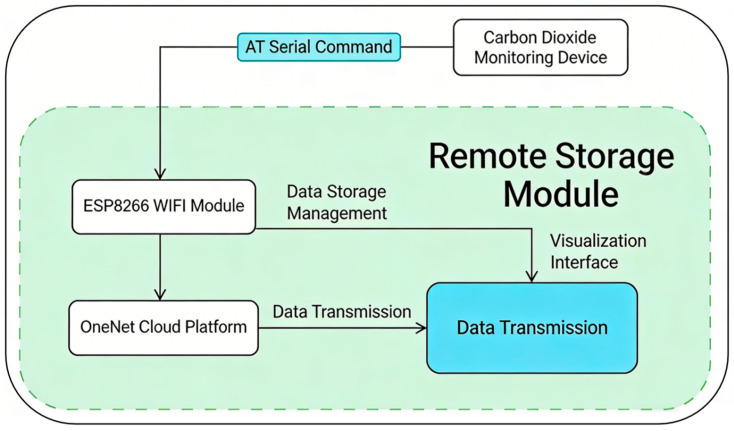
Schematic diagram of the data transmission and remote storage module. The module realizes data interaction between the carbon dioxide monitoring device and the OneNet cloud platform through AT serial commands and the ESP8266 wireless fidelity module, supporting data storage, management, and visualization.

The remote storage and monitoring module is centered on the ESP8266 Wi-Fi module. A stable connection between the computer and the monitoring device is established via this module and AT serial port commands, enabling long-term continuous data transmission [24]. As shown in Figure 7, AT serial port commands are sent to receive monitoring data, including carbon dioxide concentration, temperature, humidity, light intensity, and wind speed. These data are initially processed and packaged using the MQTT (Message Queuing Telemetry Transport) protocol and then transmitted to the OneNET cloud platform for storage. The specific data transmission process is shown in Figure 8.

**Figure 9:**
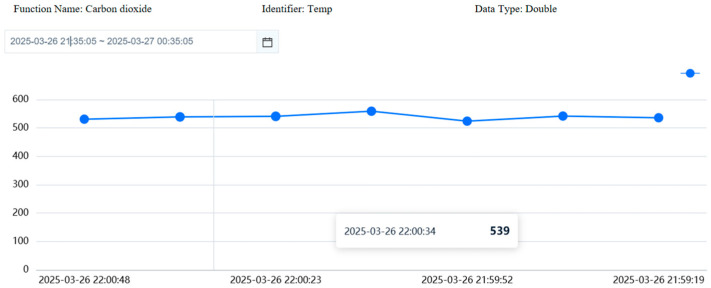
Carbon dioxide monitoring data on the OneNET cloud platform. The figure shows the time-series variation trend and specific values of carbon dioxide concentration data collected by the monitoring device and stored on the OneNET cloud platform over a continuous period.

Upon receiving data transmitted by the ESP8266, the OneNET cloud platform employs distributed storage to archive the environmental data collected from the detection devices. This data is stored in the cloud database in a time-series and sharded manner. Concurrently, the data is categorized according to time series and data type, as shown in Figure 9, to facilitate subsequent data query and analysis.

**Figure 10:**
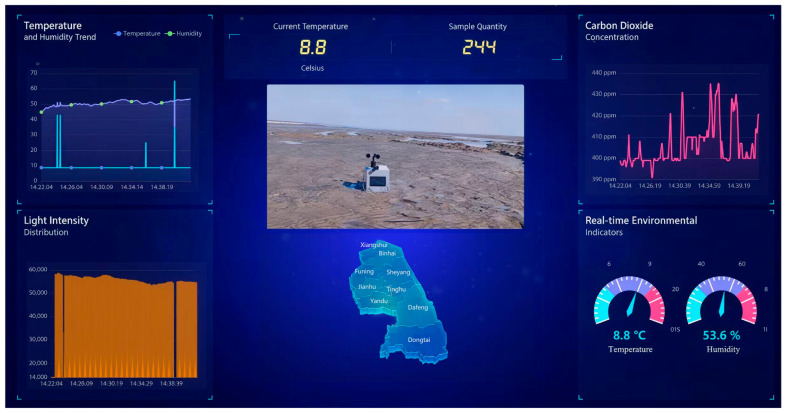
Visualization interface of the monitoring platform. The real-time interface displays the values and variation trends of temperature, humidity, light intensity and carbon dioxide concentration, and also shows the operational status of the monitoring device.

In terms of data visualization, users can view data in real time via the visual interface, as shown in Figure 10. This screen presents various data in an intuitive manner. Additionally, the visualization platform allows for the configuration of parameters such as the data display scope and time interval, as well as the format of data presentation, to accommodate the needs of different users.

### 2.3. Terminal Assessment System

The terminal assessment system employs the DNDC (DeNitrification-DeComposition) model as its core for carbon flux analysis and carbon sink evaluation. Proposed by Professor Changsheng Li’s research team in 1992, the DNDC model is a classic biogeochemical model for simulating ecosystem carbon and nitrogen cycles. It is the preferred model for related studies in the Asia-Pacific region and has been widely applied in simulating agricultural greenhouse gas emissions, wetland N_2_O emissions, and soil organic carbon dynamics [25]. However, existing research, such as Tang et al.’s study on coastal organic carbon, predominantly focuses on the current or past status of organic carbon, with limited investigations into regional organic carbon changes under future climate conditions [26].

The DNDC model comprises six sub-models, including those for soil climate and plant growth. It can predict crop growth, soil carbon dynamics, and emissions of gases such as CO_2_ and CH_4_. Supporting multi-scale simulations, the model enables large-scale carbon and nitrogen cycle analysis at the regional level when integrated with ArcGIS Pro 3.6.4. The model utilizes fundamental data such as meteorology, vegetation, and soil, integrating multidisciplinary principles to quantitatively describe carbon and nitrogen cycling. It accurately captures variations in environmental factors and dynamically predicts ecosystem carbon fluxes, meeting the complex ecological simulation requirements of salt marsh wetlands.

This study employs the DNDC model to evaluate and analyze multi-source data, including collected CO_2_ concentration, temperature, humidity, and organic carbon. As a process-driven model, it effectively simulates soil organic carbon decomposition, rhizosphere and soil respiration, and wetland greenhouse gas emission processes, while comprehensively accounting for the influence of various environmental factors. Through detailed simulations at daily and hourly scales, combined with parameters for crop growth and soil organic matter decomposition, the model precisely captures environmental dynamics and predicts CO_2_ emissions, thereby providing a clear understanding of carbon input and output patterns in wetlands.

**Figure 11:**
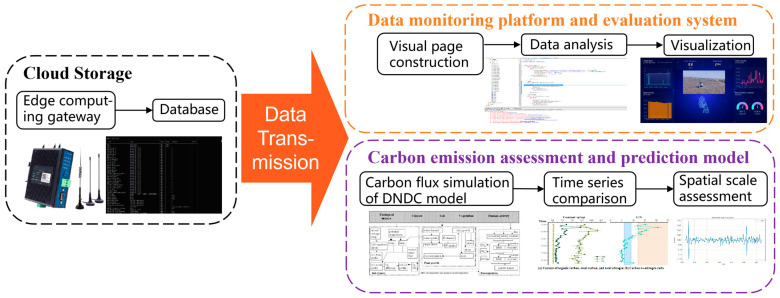
Cloud monitoring and carbon assessment system workflow. The process includes cloud data storage via an edge gateway, visualization and analysis on the monitoring platform, and DNDC model-based carbon emission assessment.

As shown in Figure 11, this study employed the DNDC model to calculate carbon fluxes at a spatial scale. Key data for the carbon cycle were extracted from a cloud database, organized, and input into the model to perform precise calculations, yielding spatial carbon flux data. Furthermore, an innovative integration of spatial and temporal sequence data was conducted to obtain more comprehensive and accurate integrated carbon flux results. This process validates the model’s applicability, provides technical guidance for carbon emission monitoring and the formulation of emission reduction strategies, and supports the achievement of carbon reduction targets.

### 2.4. Core Technologies

#### 2.4.1. Multi-Algorithm Fusion Technology Based on Spatiotemporal Coupling

**Figure 12:**
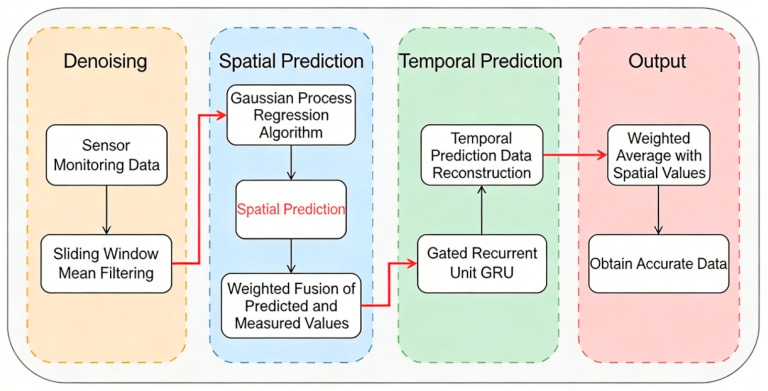
Multi-algorithm fusion technology process. The process includes four key stages, data denoising, spatial prediction, temporal prediction, and weighted fusion, to achieve accurate monitoring data.

To address the issues of insufficient accuracy and low reliability of traditional sensors in monitoring coastal salt marsh wetlands, this study innovatively proposes a multi-algorithm fusion technology based on spatiotemporal coupling to achieve high-precision CO_2_ concentration acquisition. The specific process is illustrated in Figure 12. Continuous observation data are first obtained using a self-developed spatial carbon flux monitoring device. After noise reduction via a sliding window mean filter, the Gaussian Process Regression (GPR) algorithm is employed to predict concentrations at unmonitored points, with subsequent calibration based on actual measurements. A Gated Recurrent Unit (GRU) is then integrated to construct a time-series prediction model, which is trained on historical data to forecast future concentrations. Finally, data from both spatial and temporal scales are weighted and fused to enhance overall data accuracy.

The Gaussian process regression algorithm employed for spatial prediction performs nonparametric regression based on Bayesian inference, utilizing a radial basis function (RBF) kernel [27].

For the prior distribution of the Gaussian process, it is initially assumed that the output data originate from a Gaussian process, and the joint distribution can be expressed as Formula (1).

(1) $$y\sim N(0,K\left(X,X\right)+\sigma^{2}I)$$

Here, $K\left(X,X\right)$ is the covariance matrix between known locations, $\sigma^{2}$ represents the noise term which accounts for model error, and I denotes the identity matrix.

The covariance matrix is constructed using a radial basis function (RBF) kernel, which is given by Formula (2).

(2) $$k\left(x_{i},x_{j}\right)=exp(-\frac{\left\|x_{i}-x_{j}\right\|}{{2e}^{2}})$$

For a new prediction point $x_{*}$, the joint distribution of its predicted output value $y_{*}$ and the known data y is inferred. According to the properties of Gaussian processes, this joint distribution is presented in Formula (3).

(3) $$\left[\begin{array}{c} y\\ y_{*} \end{array}\right]\sim N\left(0,\begin{array}{cc} K\left(X,X\right)+\sigma^{2}I & K\left(X,x_{*}\right)\\ K\left(x_{*},X\right) & K\left(x_{*},x_{*}\right)+\sigma^{2} \end{array}\right)$$

Here, $K\left(x_{*},X\right)$ represents the covariance vector between the known locations and the prediction point, while $K\left(x_{*},x_{*}\right)$ denotes the auto-covariance at the prediction point.

According to the inference formula for Gaussian processes, the posterior distribution is derived via conditional probability. Specifically, given the known data points X and y, the distribution of the prediction point $y_{*}$ can be obtained as shown in Formula (4).

(4) $$p\left(y_{*}|X,y,x_{*}\right)={N(K\left(x_{*},X\right)K\left(X,X\right)+\sigma^{2}I)}^{-1},K\left(x_{*},x_{*}\right)-K\left(x_{*},X\right){\left(K\left(X,X\right)+\sigma^{2}I\right)}^{-1}K\left(X,x_{*}\right)$$

Mean $\mu_{*}$, defined as the most probable predicted value, is calculated in accordance with Formula (5).

(5) $$\mu_{*}=K\left(x_{*},X\right){\left(K\left(X,X\right)+\sigma^{2}I\right)}^{-1}y$$

Variance $\sigma_{*}^{2}$, which indicates the uncertainty of prediction, is determined by Formula (6).

(6) $$\sigma_{*}^{2}=K\left(x_{*},x_{*}\right)-K\left(x_{*},X\right){\left(K\left(X,X\right)+\sigma^{2}I\right)}^{-1}K\left(X,x_{*}\right)$$

Based on the Gaussian process regression formula above, the constants in the formula are fitted using the detection values from sensors with known coordinates. This constructs a Gaussian process regression model tailored for different sensors. Subsequently, the coordinates of the unobserved points requiring prediction are input into the model, with the mean serving as the predicted value and the variance as the confidence metric.

**Figure 13:**
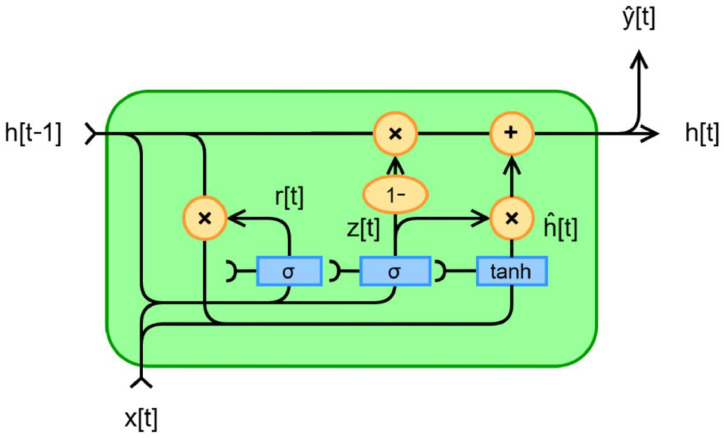
GRU computation process. The diagram illustrates the internal computation flow of the Gated Recurrent Unit, showing how the reset gate and update gate control the information flow between the previous state and current input to generate the new hidden state.

For temporal data prediction, this study integrates deep learning techniques by employing Gated Recurrent Units (GRUs) to build a time series forecasting model. Through training on historically measured spatial data and temporal replay, high-precision prediction of future carbon dioxide concentration is achieved. The GRU used for temporal prediction incorporates two core gating mechanisms: the update gate and the reset gate [28]. At time step t, the GRU computation is defined by the following formulas, with the corresponding process illustrated in Figure 13.

Update gate: This gate determines the proportion of the current hidden state derived from the previous hidden state versus the proportion derived from the current input, which is computed as Formula (7).

(7) $$z_{t}=\sigma (W_{z}\cdot \left[h_{t-1},X_{t}\right]+b_{z})$$

Here, $W_{z}$ and $b_{z}$ represent the weight matrix and bias term of the update gate, respectively.

Reset gate: This gate determines how to discard the previous state information, and its expression is given by Formula (8).

(8) $$r_{t}=\sigma (W_{r}\cdot \left[h_{t-1},X_{t}\right]+b_{r})$$

Here, $W_{r}$ and $b_{r}$ represent the weight matrix and bias of the reset gate, $h_{t-1}$ denotes the hidden state from the previous time step, and $X_{t}$ is the current input.

The optimizer employs Adam, while the loss function uses mean squared error (MSE), as specified in Formula (9).

(9) $$L=\frac{1}{n}\sum_{t=1}^{n}{\left\|{\hat{X}}_{t+k}-X_{t+k}\right\|}^{2}$$

Given the implicit relationships among carbon dioxide concentration, temperature, humidity, and light intensity, the input time series data include historical measurements of these variables. At each time step, the input is represented as a vector shown in Formula (10).

(10) $$X_{t}=[CO_{2}\left(t\right),Temp\left(t\right),Humidity\left(t\right),Light\left(t\right)]$$

The objective output is the predicted value at a future time instance, such as the data forecast for a moment k steps ahead, which is expressed as Formula (11).

(11) $${\hat{X}}_{t+k}=[\hat{CO_{2}\left(t\right)},\hat{Temp\left(t\right)},\hat{Humidity\left(t\right)},\hat{Light\left(t\right)}]$$

The vector X contains the four types of sensor information to be predicted, and the final fusion result is calculated by Formula (12).

(12) $${\tilde{X}}_{t+k}=\alpha \cdot X_{t+k}+(1-\alpha )\cdot {\hat{X}}_{t+k}$$

Finally, the predicted and the actual measured values are combined with a specific weight α to enhance prediction accuracy and system stability, as described by Formula (12).

#### 2.4.2. Spatiotemporal Scale Carbon Sink Assessment Technology Based on the DNDC Model

Traditional carbon flux monitoring primarily relies on temporal dimension analysis or long-term observations from fixed stations, which limits a comprehensive understanding of its spatial variability. This study overcomes the constraints of single-point measurements through multi-source data fusion, significantly enhancing both data coverage and accuracy. This innovative technology extends the monitoring scope from local sites to regional and even global scales, providing robust support for carbon sink policy formulation and international carbon market accounting.

**Figure 14:**
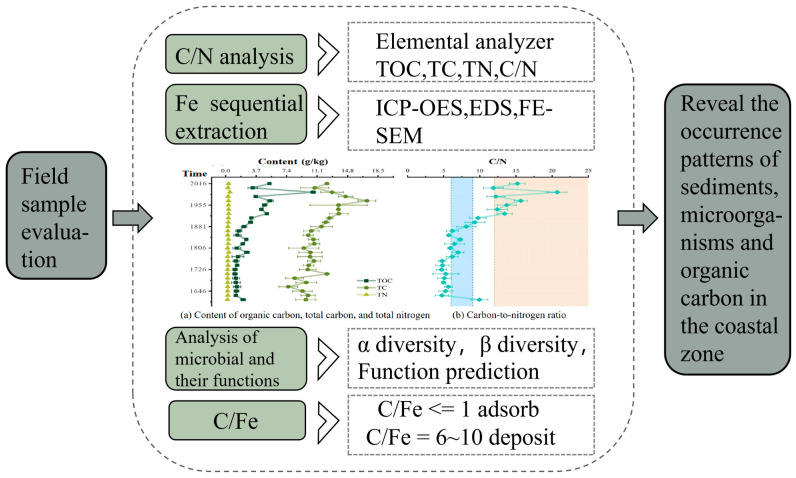
Time-series scale analysis of carbon sink evaluation. The evaluation includes sample analysis such as C/N ratio testing, iron sequential extraction, microbial functional analysis, and C/Fe ratio assessment, to reveal the occurrence patterns of sediment, microorganisms, and organic carbon in the coastal zone.

Building on this foundation, we employed the advanced DNDC model to conduct a series of critical tasks. First, to assess carbon sink patterns at the time-series scale, the DNDC model was utilized to systematically organize and deeply integrate previously measured data. These historical data encompass carbon sink variations in marine ecosystems across different time periods. Through meticulous analysis, representative patterns were extracted, laying a solid foundation for subsequent work. The design process is illustrated in Figure 14.

**Figure 15:**
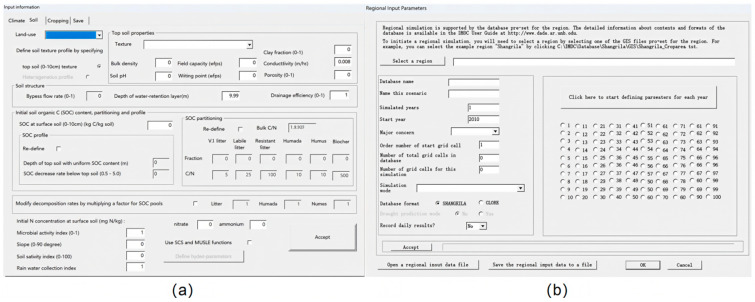
DNDC model simulation interfaces: (**a**) Point simulation program. (**b**) Area simulation program.

Subsequently, specialized carbon flux monitoring equipment was employed within a distributed network architecture and a multi-monitoring-point deployment scheme to extensively collect carbon flux data from various spatial locations in marine salt marsh wetlands. This enabled the derivation of regional carbon flux variation patterns. Based on the acquired massive dataset, the DNDC model was applied to conduct spatial-scale carbon sink assessments at both the point-specific and regional dimensions. The assessment and simulation procedure is shown in Figure 15, which facilitates precise tracking of regional carbon flux dynamics and provides data support for marine ecological conservation and climate change response.

Finally, an innovative approach integrating temporal and spatial scales was adopted. By synthesizing the temporal evolution patterns of carbon flux with its spatial distribution, a comprehensive carbon sink assessment was conducted in depth. This integration addresses the limitations of single-scale sequential data in assessing the spatial variability of marine carbon sinks, thereby reducing errors in carbon accounting.

#### 2.4.3. Innovative Land–Air Integrated Cooperative Monitoring Architecture Based on Unmanned Aerial Vehicles

**Figure 16:**
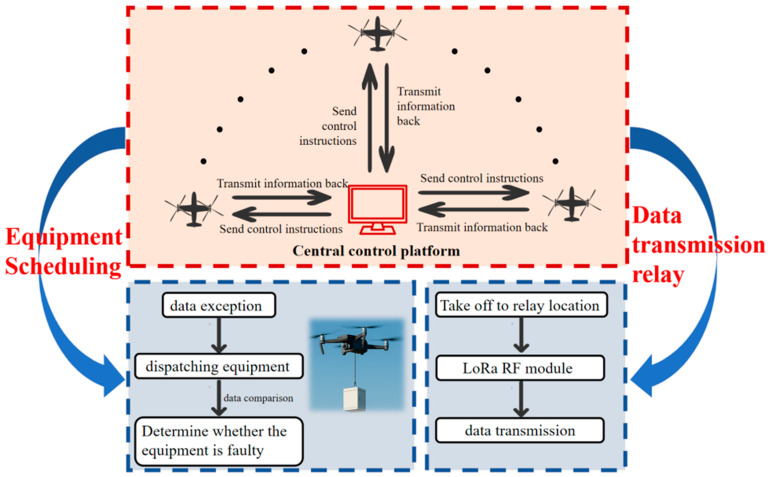
UAV-based land–air integrated cooperative monitoring architecture. This architecture supports equipment scheduling and data transmission relay via UAVs, enabling efficient wetland monitoring through a central control platform.

The coastal salt marsh wetland environment is particularly complex, making frequent manual deployment of equipment difficult. Tidal conditions can easily damage equipment, and anomalous data cannot be corrected promptly, severely compromising the accuracy and completeness of monitoring data. To address these complex challenges, this study creatively designs an innovative land–air integrated cooperative monitoring architecture specifically for salt marsh wetlands. This architecture, as shown in Figure 16, integrates multiple advanced functions to achieve efficient and reliable monitoring.

The system monitors data status in real time. When data anomalies are detected, the UAV immediately initiates task scheduling and conducts equipment inspection as a substitute. The drone features a vertical take-off and landing design, enabling rapid deployment in complex wetland environments. Upon reaching the target area, it can hover steadily near anomalous points via low-altitude hovering or cruising modes to conduct precise scanning. Its onboard backup monitoring equipment collects data in real time, compensating for gaps caused by ground equipment failure or signal interruption. Simultaneously, the system can identify issues such as sensor damage or insufficient power supply and provide targeted solutions.

**Figure 17:**
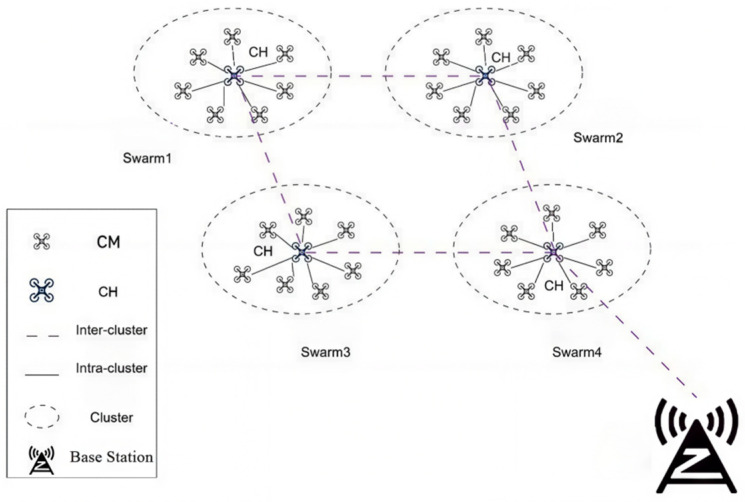
UAV swarm data transmission network framework. This clustered network architecture enables inter-swarm data relay via cluster heads, supporting encrypted transmission of monitoring data and equipment status back to the base station.

After completing data collection and fault diagnosis, the drone swarm utilizes its own network topology architecture, as shown in Figure 17, to perform data relay in physical space. It transmits real-time monitoring data, equipment status information, and on-site imagery back to the monitoring base. This process employs encrypted transmission protocols to ensure data integrity and security [29].

Upon receiving the information, the monitoring center can promptly assess the situation and decide whether to dispatch a maintenance team or adjust the monitoring strategy. This approach safeguards the continuity and reliability of wetland carbon flux monitoring. The entire response workflow is highly automated, significantly enhancing the efficiency and accuracy of ecological monitoring in salt marsh wetlands.

### 2.5. System Construction

#### 2.5.1. System Hardware Construction

**Figure 18:**
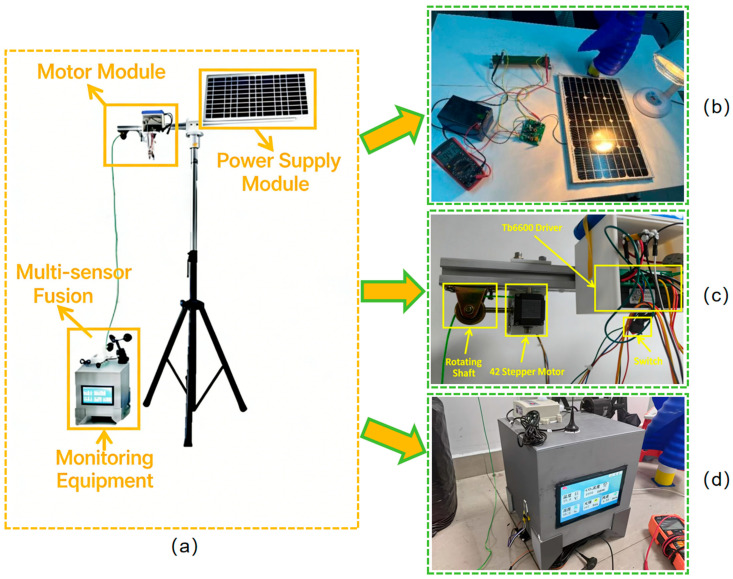
(**a**) System hardware construction. (**b**) Power supply module construction. (**c**) Motor Lifting Module Construction. (**d**) Monitoring Device Construction. (**a**) shows the integrated monitoring system, which combines the power supply module, motor lifting module, and multi-sensor monitoring equipment; (**b**) depicts the construction of the wind–solar hybrid power supply system, including the solar panel and circuit testing; (**c**) illustrates the motor lifting module, comprising the TB6600 driver, 42 stepper motor, and rotating shaft for adaptive height adjustment; (**d**) presents the assembled monitoring device with integrated multi-sensor units, communication modules, and anti-corrosion encapsulation.

Following the independent debugging of all hardware modules, on-site integration was performed. The overall hardware system, as shown in Figure 18, comprises the construction of the motor lifting module, the power supply module, and the monitoring device.

(1) Motor Lifting Module Construction

The intelligent lifting mechanism consists of a TB6600 driver, a 42 stepper motor, and 304 stainless-steel guide rails. After mechanical assembly of the motor and guide rails, an adaptive lifting program was debugged. This program automatically adjusts the sensor collection height based on tidal levels to prevent equipment from being submerged by seawater.

(2) Power Supply Module Construction

The power supply system adopts a wind–solar hybrid architecture, which includes a 200 W monocrystalline silicon solar panel, a 100 W small vertical-axis wind turbine, and a 12 V/100 Ah lithium iron phosphate battery pack. All power supply lines were constructed using waterproof and corrosion-resistant cables, achieving integrated connectivity with the monitoring device and the gateway.

(3) Monitoring Device Assembly

This study employed a self-designed PCB board as the core platform, integrating multiple monitoring units. All components were connected to the main control chip (STM32L431) via UART interfaces, enabling synchronous acquisition of multi-source data. LoRa and 4G modules were integrated both internally and on the enclosure wall of the monitoring device. After undergoing anti-corrosion encapsulation, the sensors were secured within the front-end collection chamber of the monitoring device to ensure data acquisition accuracy and stability.

#### 2.5.2. Overall Hardware and Software Integration with Field Testing

**Figure 19:**
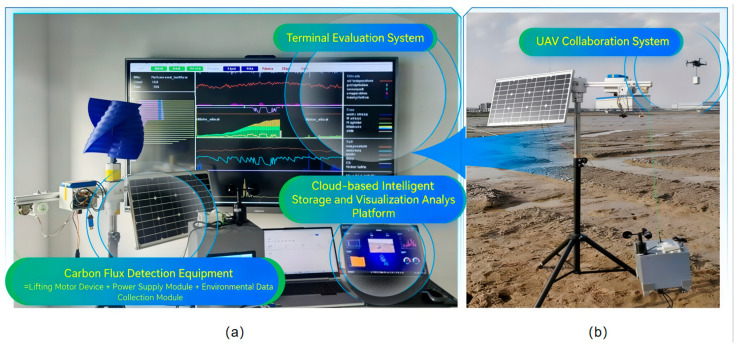
Overall hardware and software integration and field test: (**a**) Overall hardware and software integration. (**b**) Field test. (**a**) shows the fully integrated monitoring system, including the carbon flux detection equipment, cloud-based intelligent storage and visualization analysis platform, and terminal evaluation system; (**b**) presents the field test setup with UAV collaboration, deployed at the Yancheng Dafeng wetland for on-site operation.

The monitoring device and the edge gateway formed a network via LoRa modules. The gateway then communicated with the cloud platform through a 4G module, facilitating local data processing and remote transmission. Subsequently, the system, along with a coordinated UAV system, was deployed for continuous operation in a salt marsh wetland field site. This deployment aimed to validate hardware stability and data acquisition reliability under high salinity and humidity, and tidal conditions, while also optimizing sealing and mounting structures. The testing setup is shown in Figure 19.

## 3. Results

### 3.1. System Output Sensor Monitoring Results

The CO_2_ measurement in this system utilizes an indirect method, wherein an NDIR sensor monitors changes in atmospheric CO_2_ concentration at 10 cm above the ground surface, rather than directly measuring the CO_2_ flux from the sediment. The net carbon uptake of a wetland ecosystem cannot be directly derived from a single concentration value; instead, it is estimated by combining continuous monitoring of diurnal CO_2_ concentration variations with a nocturnal respiration baseline [30]. Specifically, the system measures the ecosystem respiration flux under stable nighttime conditions as a baseline. This baseline is then combined with the rate of daytime CO_2_ concentration decline, applying a difference method to estimate the amount of carbon fixed by vegetation photosynthesis, thereby indirectly reflecting the net ecosystem carbon exchange.

To eliminate interference from environmental factors on CO_2_ concentration measurements, the following control and correction measures were implemented in this study. First, the system simultaneously collected environmental parameters such as wind speed, temperature, humidity, and light intensity, and flagged and excluded data segments under abnormal meteorological conditions (e.g., instantaneous wind speed exceeding 8 m/s, temperature above 40 °C, rainfall periods, and sensor immersion during high tide). Second, a multi-algorithm fusion model incorporated temperature, humidity, and light intensity as input features in time-series prediction, effectively decoupling the interference of meteorological fluctuations from CO_2_ concentrations. Third, data used for carbon sink accounting were prioritized from stable monitoring windows with wind speeds below 3 m/s, no rainfall, and tidal levels lower than the sensor height. Fourth, through multi-point distributed deployment (four monitoring sites) and long-term continuous observation (over 24 h), spatial and temporal averaging is applied to suppress local turbulence and random disturbances. These measures together ensure that observed CO_2_ concentration variations primarily reflect the ecosystem’s carbon exchange processes rather than environmental noise.

This study implemented a distributed networking scheme with four devices to conduct a 24 h continuous monitoring experiment, verifying the reliability of multi-sensor data fusion technology in a marine salt marsh wetland environment. During the experiment, the system simultaneously collected four sets of raw monitoring data at one-minute sampling intervals, yielding a total of 5744 valid data points. To enhance data quality, a sliding window moving average filter algorithm was first applied to preprocess the raw data, effectively removing environmental noise and equipment interference. Building on this, a spatiotemporal coupling fusion model incorporating Gaussian Process Regression (GPR) and Gated Recurrent Units (GRUs) was introduced for in-depth data processing and outlier identification.

Following algorithmic processing, 16 sets of anomalous data were identified, as shown in Table 1. The overall data qualification rate reached 99.72%, which robustly demonstrates the monitoring stability and data reliability of the system within the complex wetland environment. This outcome indicates that the multi-sensor data fusion technology can effectively overcome the limitations of traditional sensors in salt marsh wetland monitoring, such as insufficient precision and low data reliability, thereby establishing a solid data foundation for subsequent carbon flux assessment.

**Figure 20:**
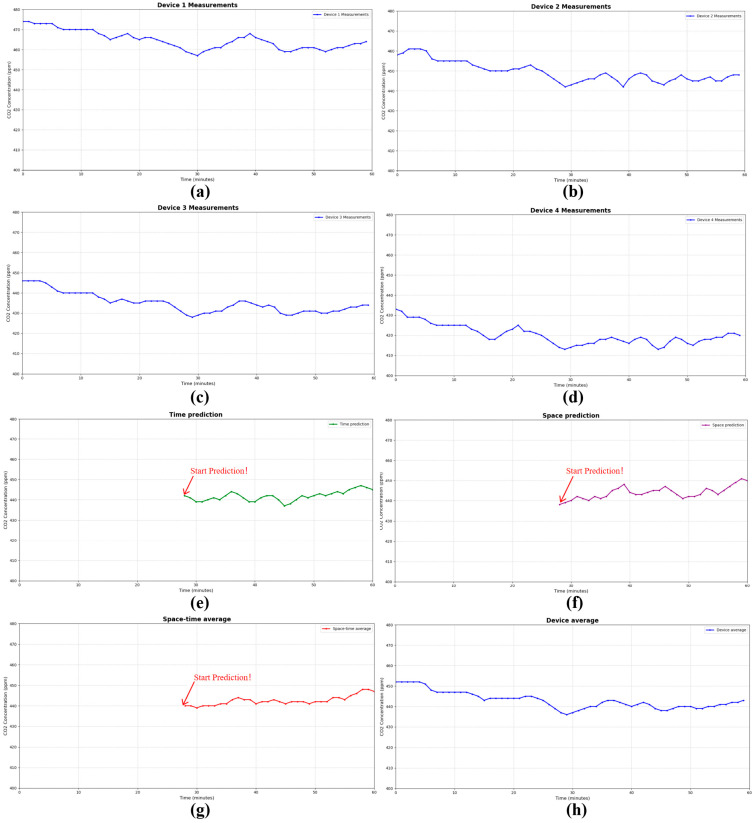
Actual monitoring and prediction curves of CO_2_ concentration. (**a**–**d**) show the measured values from Device 1 to Device 4, respectively; (**e**) is the time prediction, (**f**) is the space prediction, (**g**) is the spatiotemporal coupling prediction, (**h**) represents the average monitoring value of the four devices.

The multi-algorithm fusion technique also enables the prediction of carbon flux data over short timeframes, as demonstrated by the monitoring and forecasting results for carbon dioxide concentration within one hour after training shown in Figure 20. A comparison of the curves reveals that the predicted values generated by the fusion algorithm closely align with the on-site measured values, with the relative error controlled within 0.5%. This indicates that the technique can effectively reconstruct the dynamic trends of environmental parameters. Furthermore, by training on historical data and performing temporal replay, the system can achieve high-precision predictions of future carbon dioxide concentrations, thereby enhancing the practical utility of the monitoring data.

### 3.2. Carbon Sink Projections from the DNDC Model Output

**Figure 21:**
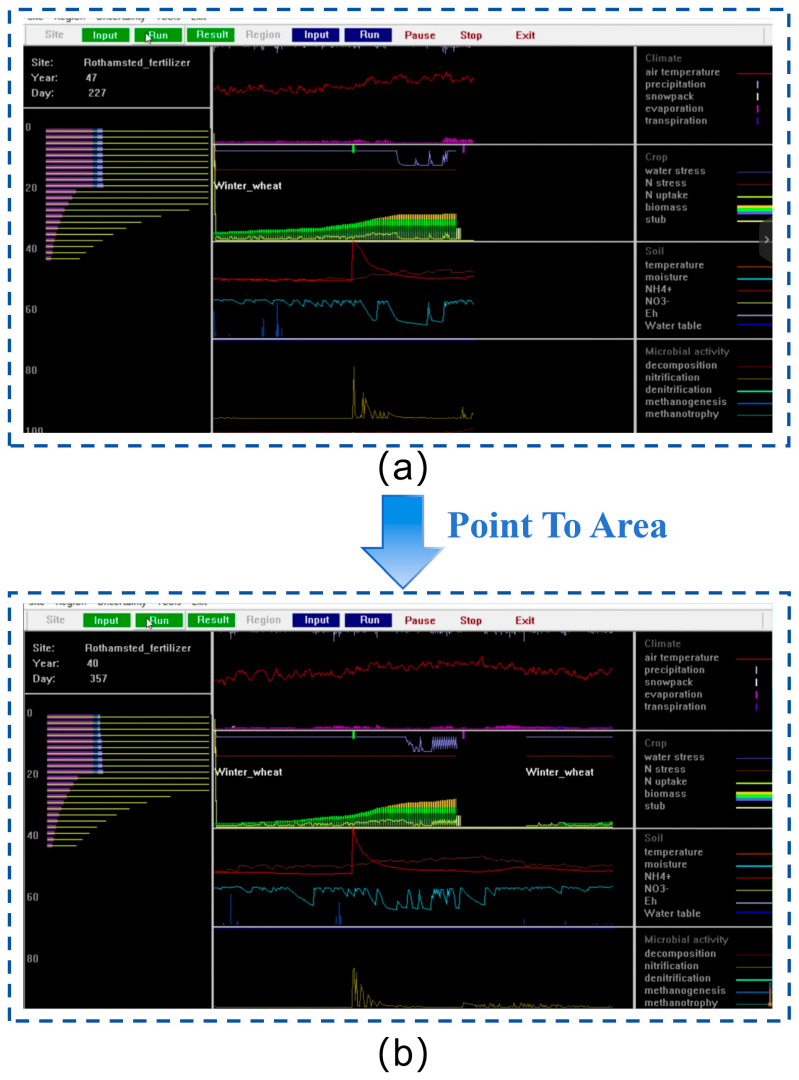
Process of the DNDC model: (**a**) Model run of point. (**b**) Model run of area. To validate the DNDC-based spatiotemporal carbon sink assessment, this study conducted point-scale calibration first using field-measured data from typical monitoring sites, then extrapolated the calibrated parameters to the regional scale for carbon flux simulation across the 100-hectare wetland.

To validate the effectiveness of the spatiotemporal-scale carbon sink assessment technology based on the DNDC model, this study selected a 100-hectare coastal salt marsh wetland in Dafeng District, Yancheng City as the research area. Focusing on the annual carbon sink dynamics across twelve months, multi-source data including soil properties, vegetation types, and meteorological conditions from cloud databases, along with CO_2_ concentration, temperature, and humidity collected by monitoring equipment, were standardized and input into the DNDC model for carbon sink prediction and analysis. The operational process of the DNDC model is shown in Figure 21. The research adopted a progressive approach of “point-scale simulation to regional extrapolation.” First, model parameters were locally calibrated using measured data from typical monitoring points. Then, the study area was divided into grid units using ArcGIS to simulate and predict carbon fluxes and carbon sink amounts at the regional scale. This enabled a systematic analysis of the spatiotemporal distribution characteristics and evolution patterns of carbon sinks in the salt marsh wetland.

#### 3.2.1. Carbon Sequestration Prediction Results at the Point Scale

Based on continuous monitoring data from January to December, carbon sequestration dynamics were predicted at the point scale for four typical monitoring sites within the study area. These sites included a nearshore intertidal zone, a middle tidal zone spartina alterniflora area, an offshore low-salinity zone, and a phragmites australis-suaeda salsa mixed area. The results are presented in Table 2.

As shown in Table 2, the middle tidal zone spartina alterniflora area exhibited the strongest carbon sequestration capacity among all monitoring sites, with an annual cumulative carbon sink of 1935.1 g C/m^2^. Its monthly average sequestration intensity was approximately twice that of the nearshore intertidal zone, highlighting the carbon fixation advantages conferred by the high photosynthetic efficiency and well-developed root systems of spartina alterniflora. All sites displayed pronounced seasonal variations in carbon sequestration intensity. Carbon sequestration peaked in August, driven by vigorous vegetation photosynthesis and high soil microbial activity, while it reached its lowest point in January due to low temperatures and vegetation senescence. The coefficient of determination (R^2^) between model-predicted and measured values exceeded 0.87 at all sites. This indicates that the DNDC model, after calibration with localized parameters, can accurately capture the dynamics of carbon sequestration at the site scale in marine salt marsh wetlands, yielding highly reliable predictions.

#### 3.2.2. Carbon Sequestration Prediction Results at the Regional Scale

The 100-hectare study area was divided into 100 m × 100 m grid cells (totaling 100 cells) using ArcGIS. Regional-scale carbon sequestration predictions were conducted based on the site-calibrated parameters to clarify the spatial distribution characteristics of carbon sequestration. The results are presented in Table 3.

The regional-scale prediction results indicate that the total annual carbon sequestration in the 100-hectare marine salt marsh wetland study area reached 1498.4 t C, with an average carbon sequestration per unit area of 1498.4 g C/m^2^, demonstrating an efficient blue carbon ecosystem overall. The spatial heterogeneity of carbon sequestration capacity was significant. Although the middle tidal zone spartina alterniflora area accounted for only 42.5% of the total study area, its contribution to carbon sequestration was as high as 54.9%, identifying it as the core carbon sink region. In contrast, the nearshore intertidal zone exhibited the weakest carbon sequestration capacity, with its cumulative carbon sequestration per unit area being less than 50% of that in the middle tidal zone spartina alterniflora area. This weakness is attributed to frequent tidal scouring, high soil salinity, and low vegetation coverage. The carbon sequestration contribution characteristics of each zone align closely with the actual ecological and environmental conditions of the study area, demonstrating that the DNDC model can effectively achieve accurate carbon sequestration predictions at the regional scale for marine salt marsh wetlands.

#### 3.2.3. Validation of Spatiotemporal Coupling Carbon Sink Prediction Effectiveness

The spatial-scale carbon sink data predicted by the DNDC model were integrated with the temporal evolution patterns of carbon sinks from January to December. Compared to single-scale assessment methods, this integration validated the effectiveness of the spatiotemporal coupling prediction approach. The results are presented in Table 4.

The measured total carbon sink in the study area was 1421.7 t C. The prediction based solely on time-series assessment, which did not account for spatial heterogeneity in carbon sinks, was lower than the measured value, with an error of −8.5%. Conversely, the prediction based solely on spatial-scale assessment, which failed to reflect seasonal dynamics of carbon sinks, was higher than the measured value, with an error of 12.8%. In contrast, the spatiotemporal coupling prediction method developed in this study integrates full spatial distribution and annual temporal dynamics, thereby overcoming the inherent limitations of single-scale assessments. This method achieved an error of only 5.1% compared to the measured value, while providing 100% spatial coverage and complete temporal continuity over 12 months. These results demonstrate that this technique can effectively enhance the accuracy and comprehensiveness of carbon sink accounting for coastal salt marsh wetlands, offering a reliable methodological foundation for the scientific assessment of blue carbon sink potential.

## 4. Discussion

Traditional carbon flux monitoring primarily relies on chamber-based and gradient-based techniques. The static chamber method is cost-effective, but chamber deployment can disrupt microenvironmental airflow and does not support continuous monitoring, typically yielding fewer than six valid data points per day [31]. The dynamic chamber method mitigates gas accumulation effects; however, mechanical pump vibrations alter soil pore structure, leading to an overestimation of flux values by 15% to 30% [32]. Gradient methods, represented by eddy covariance systems, offer higher accuracy but depend on complex turbulence theory. They require the installation of towers ranging from 10 to 40 m in height for high-frequency monitoring, with a single system costing over $100,000. Furthermore, these methods demand stringent surface homogeneity and can incur errors exceeding ±25% when applied in heterogeneous environments such as intertidal zones [33]. Features of some similar products are presented in Table 5.

Compared with existing mainstream methods in the field of salt marsh wetland carbon flux monitoring, the proposed system demonstrates significant advantages in both performance and costs. Conventional static chamber–gas chromatography and eddy covariance systems exhibit notable limitations: the static chamber method is susceptible to environmental disturbances, often yielding relative errors in flux estimates ranging from 15% to 30% [34]; whereas eddy covariance equipment entails high costs and complex maintenance, and is prone to data biases over heterogeneous substrates such as salt marshes [35]. Although commercial nondispersive infrared (NDIR) gas analyzers offer high precision, they demonstrate poor adaptability to the high salinity mist and humidity conditions characteristic of salt marshes, suffering from zero-point drift that necessitates frequent calibration [36]. In contrast, the monitoring system developed in this study confines the relative error of gas concentration measurements to within 0.31%, reduces comprehensive equipment costs by more than 60%, maintains a stable performance under high-salinity conditions, and substantially diminishes the need for manual intervention. Its lightweight, multi-algorithm fusion framework streamlines data processing workflows, rendering it particularly suitable for long-term, unattended monitoring within the complex ecosystems of salt marshes.

This study addresses the core challenges in monitoring carbon fluxes in salt marsh wetlands through systematic innovation, demonstrating significant advantages over traditional methods and comparable products. Technologically, it develops a multi-sensor data fusion technique integrating Gaussian Process Regression (GPR) and Gated Recurrent Units (GRUs). Through filtering, noise reduction, spatiotemporal data extrapolation, and weighted fusion, the fluctuation range of monitoring data is reduced to 0.5%, substantially enhancing data accuracy. Architecturally, a dedicated integrated land–air collaborative monitoring framework is designed. This framework incorporates intelligent lifting devices, a wind–solar hybrid power supply system, and drone swarms, adapting to the complex salt marsh environment to achieve over 72 h of uninterrupted endurance and long-term automated monitoring. Methodologically, a spatiotemporally coupled carbon sink assessment system based on the DNDC model is constructed. This system overcomes the limitations of single-scale approaches by integrating spatially distributed data with temporal sequence patterns, resulting in a mere 5.8% error between carbon sink accounting and measured values. In terms of cost and adaptability, a fully self-developed solution with optimized design is adopted, reducing costs by over 60% compared to similar products. The hardware undergoes anti-corrosion and waterproof treatment, achieving a monitoring data qualification rate of 99.72%. This represents comprehensive optimization in precision, adaptability, and cost-effectiveness.

Although this system demonstrates a satisfactory performance in monitoring carbon fluxes in salt marsh wetlands, it still presents several application limitations and uncertainties. First, under extreme weather conditions such as strong typhoons and heavy rainfall, drone inspection missions may be interrupted, compromising the continuity of data collection and indicating that the system’s environmental adaptability requires further improvement. Second, the parameterization of the DNDC model for different salt marsh vegetation types has not yet been fully localized, and model outputs remain sensitive to vegetation parameters, leading to uncertainties in regional upscaling applications. Finally, the stability of the system during long-term field operation has not been sufficiently validated. Under high-salinity and foggy environments, the corrosion resistance of equipment and the reliability of data acquisition still need to be assessed through more extended continuous observations. Future efforts will focus on further reducing system uncertainty through upgrades to hardware protection, optimization of model parameters, and long-term stationary monitoring.

## 5. Conclusions

This study addresses the challenges of poor equipment adaptability, low data accuracy, and single-scale carbon sink assessment in salt marsh wetland carbon flux monitoring by independently developing a spatiotemporally coupled carbon flux monitoring system. The system integrates GPR-GRU multi-algorithm fusion technology, constraining monitoring data fluctuations within 0.5% and achieving a spatiotemporal comprehensive prediction relative error of merely 0.31%. Through an air–ground collaborative monitoring architecture, the system enables long-term automated operation under complex environmental conditions, with a data qualification rate reaching 99.72%. Furthermore, the spatiotemporally coupled carbon sink assessment method based on the DNDC model reduces carbon sink accounting errors to 5.1%, significantly enhancing assessment precision.

The core contributions of this research are manifested in three aspects: First, it proposes a multi-algorithm sensor data correction method fusing GPR and GRU, which controls monitoring data fluctuations within 0.5% and limits the spatiotemporal comprehensive prediction relative error to 0.31%, effectively improving monitoring accuracy in the complex environments of salt marshes. Second, an integrated air–ground collaborative monitoring architecture tailored for salt marsh wetlands was constructed. By incorporating intelligent lifting mechanisms, hybrid wind–solar power supply, and unmanned aerial vehicle (UAV) inspection capabilities, the system achieves long-term automated operation with a data qualification rate of 99.72%. Furthermore, comprehensive equipment costs are reduced by over 60%, substantially diminishing the need for manual intervention. Third, a spatiotemporal coupled carbon sink assessment framework based on the DNDC model was established. This approach reduces carbon sink accounting errors from 8.5% and 12.8% under single-scale assessments to 5.1%, thereby addressing the limitations inherent in traditional evaluations that rely solely on either temporal sequences or spatial scales.

Nevertheless, further verification is required regarding the system’s operational stability under extreme weather conditions, the adaptability of DNDC model parameters to diverse vegetation types, and its long-term corrosion resistance in field environments. Future work will focus on refining the aforementioned aspects and further verifying the system’s applicability through long-term cross-regional observations.

## Figures and Tables

**Table 1:** Validation results for system monitoring and predictive performance using multi-algorithm fusion.

Validation Item	Results	Analysis
Overall Performance	5744/5760 successful; success rate: 99.72–95% CI: [99.65%, 99.79%]	Standard error: 0.07%
Abnormal Data Statistics	Sensor timeout: 4 cases (26.67%); Data over-limit: 5 cases (33.33%); Communication timeout: 4 cases (26.67%); object slippage: 3 (20%)	Total failures: 16 (0.28%)
Spatiotemporal Prediction Accuracy	Spatial distribution prediction relative error: 0.28%; Time series prediction relative error: 0.35%; Comprehensive prediction relative error: 0.31%	RMSE (Root Mean Square Error): 0.31 ppm; No significant effect
Technical Validation	Sampling frequency: 1 time/min; Network scale: 4; Environmental adaptability: Tolerant to high salinity, high humidity and tidal environment of salt marsh wetland	System robustness confirmed

**Table 2:** Carbon Sequestration Prediction Results at the Point Scale (January to December).

Monitoring Points	Cumulative Carbon Sink (g C/m^2^)	Monthly Average Carbon Sink Intensity (g C/m^2^·Month)	Maximum Value (g C/m^2^·Month, August)	Minimum Value (g C/m^2^·Month, January)	Fitting Degree with Measured Values (R^2^)
Nearshore Intertidal Zone	810.7	67.6	102.7	38.2	0.87
Middle Tidal Zone Spartina alterniflora Area	1935.1	161.3	215.3	56.8	0.91
Offshore Low-Salinity Zone	1262.2	105.2	138.5	45.9	0.88
Phragmites australis-Suaeda salsa Mixed Area	1188.9	99.1	126.8	39.7	0.89

**Table 3:** Regional-scale carbon sequestration prediction results (January to December).

Regional Types	Area Proportion (%)	Cumulative Carbon Sink Total (t C)	Cumulative Carbon Sink per Unit Area (g C/m^2^)	Regional Carbon Sink Contribution Rate (%)
Nearshore Intertidal Zone	7.5	60.8	810.7	4.1
Middle Tidal Zone Spartina alterniflora Area	42.5	822.4	1935.1	54.9
Offshore Low-Salinity Zone	28.3	357.2	1262.2	23.8
Phragmites australis-Suaeda salsa Mixed Area	21.7	258.0	1188.9	17.2
Entire Study Area	100.0	1498.4	1498.4	100.0

**Table 4:** Validation results for spatiotemporal coupling carbon sink prediction effectiveness.

Assessment Methods	Predicted Total Carbon Sink (t C)	Error with Measured Total Carbon Sink (%)	Spatial Coverage (%)	Temporal Continuity (Months)
Spatiotemporal Coupled Prediction (This Study)	1498.4	5.1	100	12
Time-Series Only Assessment	1301.5	−8.5	35.2	12
Spatial-Scale Only Assessment	1604.1	12.8	100.0	1

**Table 5:** Analysis of the Advantages and Disadvantages of Comparable Carbon Flux Monitoring Products.

Device Name	Advantage	Disadvantage
Vorticity Covariance System	1. High precision	1. Expensive
	2. Wide applicability	2. High site requirements
	3. International standardization	3. Complex Maintenance
LI-COR LI-8100A	1. Strong portability	1. Expensive
	2. Easy to operate	2. High site requirements
	3. High sensitivity	3. Poor portability
Picarro G2508	1. Ultra-high precision	1. Expensive
	2. Synchronous measurement of multiple gases	2. Indirect flux measurement
	3. Long-term stability	3. Poor portability
Campbell EC150	1. Low cost	1. Sensitive To the weather
	2. Strong durability	2. Limited precision
	3. Good real-time performance	3. Frequent maintenance
LGR Ultra-Portable analyzer	1. Excellent portability	1. Expensive
	2. High precision	2. Indirectness of flux
	3. Flexible scenarios	3. Limited battery life

## Data Availability

The original contributions presented in the study are included in the following publicly available repository: https://github.com/Zha20050719/Monitoring-database.git (accessed on 15 March 2026).

## Abbreviations

The following abbreviations are used in this manuscript:

DNDC	DeNitrification-DeComposition
UPS	Uninterruptible Power Supply
UAV	Unmanned Aerial Vehicle
LoRa	Long Range
4G	4th-Generation Mobile Communication Technology
MQTT	Message Queuing Telemetry Transport
IP67	Ingress Protection 67
RMSE	Root Mean Square Error
MSE	Mean Squared Error
